# Giant thoracic hematoma post-transradial coronary angiography: a case report and review of the literature

**DOI:** 10.1186/s12872-023-03466-7

**Published:** 2023-09-07

**Authors:** Ke Wang, Li Wen, Li Xie, Maoyu Zhao, Xi Liu, Xiaolin Luo, Jun Jin, Zhexue Qin

**Affiliations:** 1 Department of Cardiology, Xinqiao Hospital, Army Medical University, Chongqing, China; 2 Department of Cardiology, The First Affiliated Hospital of Chongqing Medical University, Chongqing, China

**Keywords:** Thoracic hematoma, Access complications, Transradial approach, Case report, Literature review

## Abstract

**Background:**

Although there are cardiac interventional procedures, certain transradial access complications might be life-threatening.

**Case presentation:**

A 67-year-old male was admitted for coronary angiography due to chest tightness and shortness of breath on exertion. Hours after the right transradial access angiography, the patients complained the right side of chest pain. Emergent chest X-ray revealed a giant mass in the right chest. The right radial artery was reaccessed and subsequent arteriograms confirmed that the presence of a rupture of the branch of right internal mammary artery. Simultaneously, a microcoil was implanted to seal the perforation. The perforation caused a thoracic hematoma measuring 13.8 cm × 6.7 cm, along with a decrease in hemoglobin concentration from 14.1 g/dL to a minimum of 7.8 g/dL. Additionally, the drainage of the hematoma and red blood cells transfusion were carried out. Further, the patient underwent ascending aortic replacement, aortic valve replacement, mitral valve replacement, and thoracic hematoma removal. Postoperative echocardiography showed that the prosthetic valves were properly positioned and functioning normally. The patient recovered well after the surgery and remained event-free during the latest 14moth follow-up period.

**Conclusions:**

Vascular perforation and subsequent hematoma might occur due to guidewire maneuvering during transradial approach. Awareness of prevention, early recognition and management of access complications may help reduce the occurrence and severity of complications related to the transradial approach.

## Background

The transradial approach (TRA), an alternative to the transfemoral approach, is widely used for coronary angiography (CAG) and percutaneous coronary intervention (PCI). Compared to the transfemoral approach, the TRA is related to a lower risk of access site-related complications and even mortality in high-risk PCI patients [1, 2]. Based on growing evidence, it was highly preferred by interventional cardiologists and was recommended by the European Society of Cardiology and the American Heart Association for invasive evaluation for patients with acute coronary syndrome [3, 4].

However, TRA might still lead to rare and serious access-related complications, including subclavian vessel dissection and vessel perforation in the brachiocephalic artery, internal mammary artery, and thyrocervical trunk. Given the possible life-threatening consequences, caution should be taken when carrying out the procedures. Herein, we report a case of giant thoracic hematoma caused by perforation of the right internal mammary artery (RIMA) during transradial coronary angiography and its subsequently successful management. Moreover, we reviewed transradial access-related perforations and other complications.

## Case presentation

A 67-year-old male smoker with a history of chronic obstructive pulmonary disease, hypertension and diabetes mellitus was admitted to our department due to chest tightness and shortness of breath on exertion. The electrocardiogram demonstrated normal sinus rhythm with nonspecific ST-T-wave changes. Transthoracic echocardiography (TTE) revealed an enlarged left atrium (46 mm) and left ventricle (58 mm), widened sinus of Valsalva (45 mm) and ascending aorta (42 mm), severe regurgitation of mitral (regurgitation arear 10.4cm^2^ per beat) and aortic (regurgitation arear 11.7cm^2^ per beat) valves, and left ventricular systolic ejection fraction of 57%.

For the coronary evaluation, CAG was performed under fluoroscopic guidance, using the right radial artery, a 6 French (Fr) sheath, a 0.035 inch ×150 cm, angled, J-type Radifocus hydrophilic guidewire (Terumo, Tokyo, Japan), and a 5 Fr radial diagnostic catheter (TIG 110 cm, Angiopointer, Hunan, China). The hydrophilic wire was inadvertently advanced into RIMA, and its tip transiently entered a small branch during the exchange for a Judkins right catheter (3.5 curve, 100 cm, Cordis, USA), because the TIG failed to engage the ostium of the right coronary artery. CAG showed no significant coronary stenosis.

**Fig. 1:**
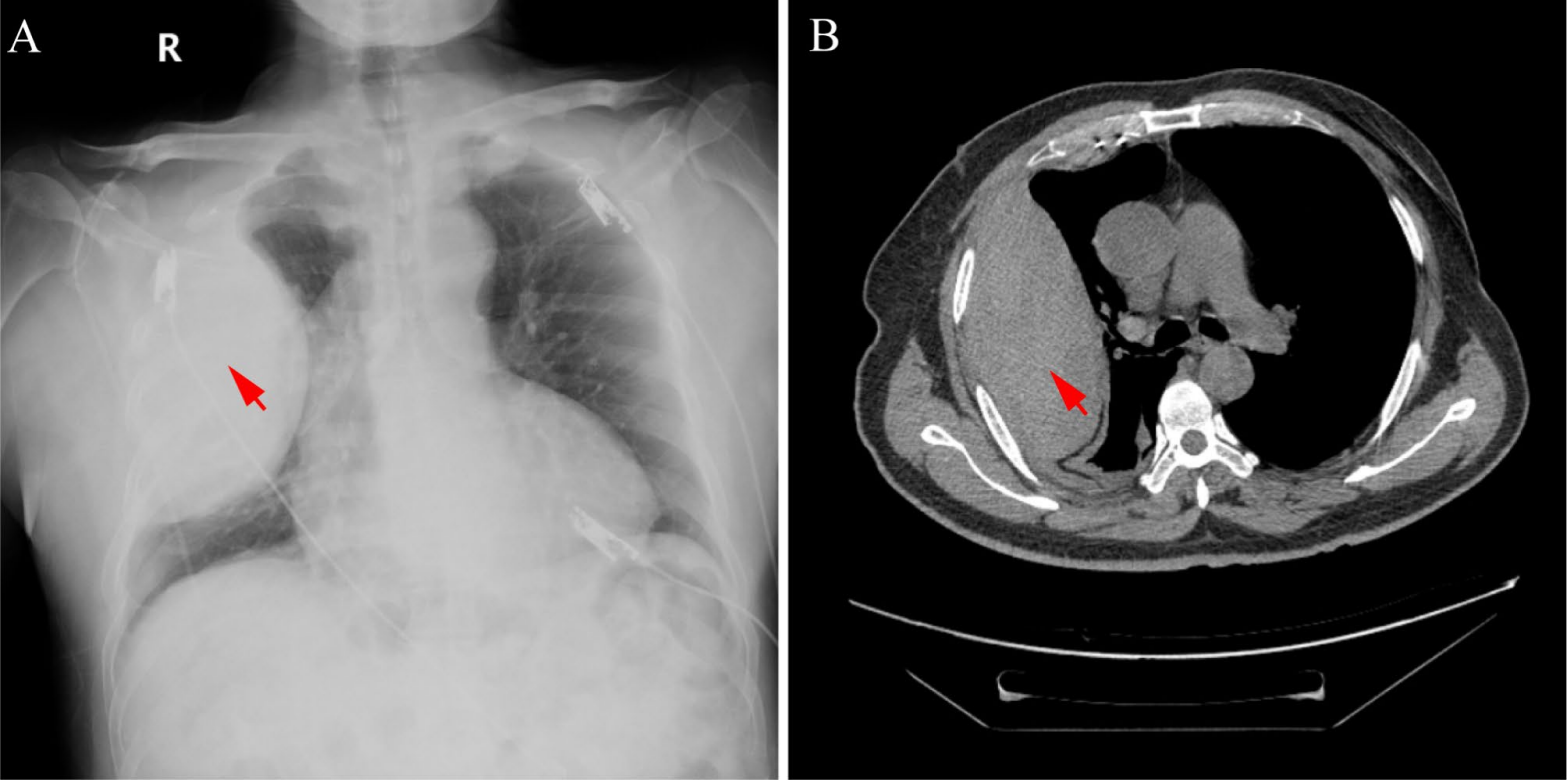
Radiographic images of encapsulated pleural effusion (red arrow)

Hours after the procedure, the patient complained of paroxysmal pain in the right side of the chest. The vital signs were stable and no significant differences in the results from chest physical examination as well as immediate electrocardiogram, TTE and cardiac enzyme profile were found compared with those before the procedure. Latently, the hemoglobin level fell from 14.1 to 12.7 g/dL. Emergent chest X-ray results revealed a new mass in the right outer lung area suspected to be an encapsulated pleural effusion (Fig. 1A). The branch of thoracic artery perforation was highly suspected, and computed tomography angiography of the access vessels was performed. The specific arterial bleeding site was scrutinized and not observed for the poke-shaped encapsulated effusion in the right pleural cavity (13.8 cm × 6.7 cm) (Fig. 1B).

**Fig. 2:**
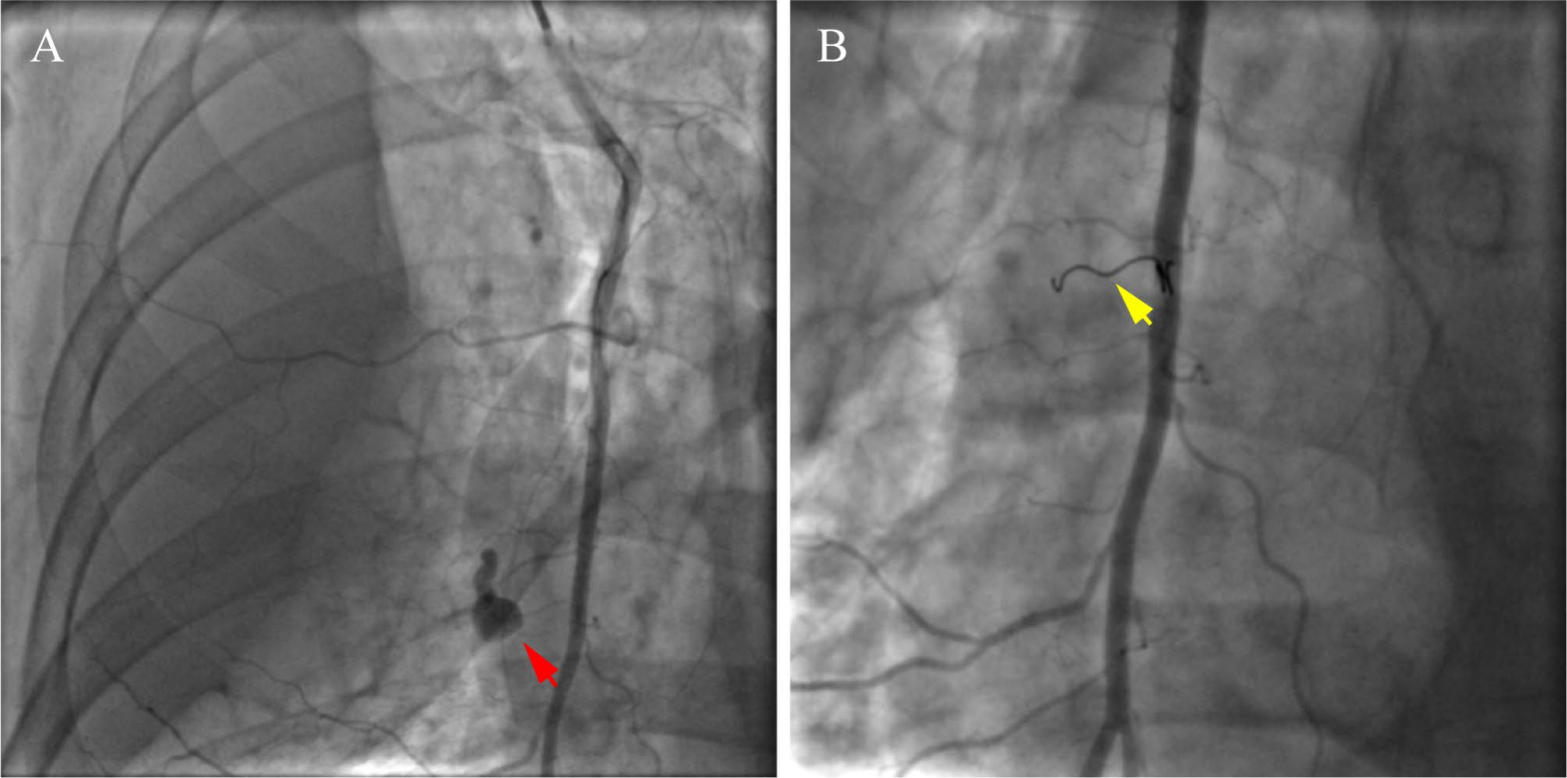
Angiographic images of the perforation (red arrow) before **(A)** and after **(B)** embolization with the microcoil (yellow arrow)

Precautious volume supplementation was performed and the patient was taken to the catheterization laboratory again for further scrutinization. Right radial access was taken again and the TIG was used for right subclavian and internal mammary arteriograms. The right subclavian arteries were intact, whereas contrast extravasation was noted in the terminal portion of the branch from RIMA (Fig. 2A). Then a 6 Fr right Judkins guiding catheter was used to engage the RIMA ostium. Endovascular occlusion therapy was performed successfully by delivering a Tornado embolization microcoil (Cook Medical, Bloomington, IN, USA) via the Finecross microcatheter (Terumo, Tokyo, Japan) to the perforated branch. Angiogram confirmed that the extravasation was extinguished (Fig. 2B).

Later, thoracic drainage was performed to relieve pulmonary compression due to extravasation. In total, 5.4 L of bloody pleural effusion was drained from the hemothorax. The drainage was removed after 12 days, when drainage was minimal. The hemoglobin concentration gradually decreased to a minimum of 7.8 g/dL and two units of packed red blood cells were transfused. The hemoglobin levels finally stabilized at approximately 10.2 g/dL after 15 days.

**Fig. 3:**
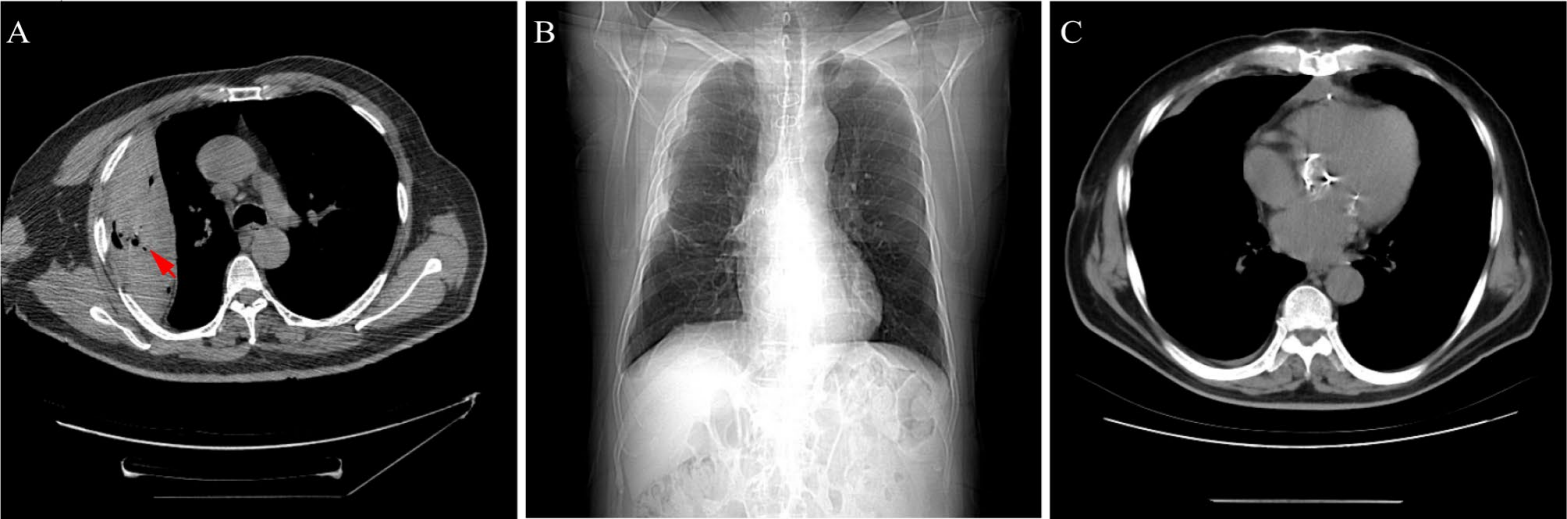
Images of encapsulated pleural effusion during treatment. **A**. Image of encapsulated pleural effusion (red arrow) after embolization and drainage treatment. **B**&**C**. Images after surgery

After 4 weeks of optimized treatment, the dyspnea of the patient was not improved significantly. Furthermore, the pulmonary function test was performed. The forced expiratory volume in one second (FEV1) and the forced vital capacity (FVC) were measured. It was predicated that the patient had coexisting severe obstructive and restrictive lung disease with the FEV1/FVC ratio of 46.22%, FEV1 of 29.6%, and FVC 50.9%. Chest Computed Tomography (CT) showed cavities in the encapsulated pleural effusion (Fig. 3A).

After full discussion by the expert committee composed of specialists from cardiac surgery, thoracic surgery and cardiology, combined surgeries of valve replacement and hematomaectomy were recommended for the patient. Subsequently, the patient underwent ascending aortic replacement, AVR, MVR, and thoracic hematoma removal using cardiopulmonary bypass (CPB). The total CPB time was 284 min, and the aortic cross-clamp time was 197 min. He was extubated within 24 h of surgery. The postoperative course was uneventful. Postoperative hemostatic vasoactive agents (dopamine, epinephrine, etc.), and antibiotics were routinely applied. Hematoma was completely removed as presented in postoperative chest CT (Fig. 3B&C). Postoperative TTE revealed that the prosthetic valves were well-seated and functioning normally. The patient was discharged on the 20th post-surgery day. During the 14-month follow-up, the patient presented no further chest discomfort or dyspnea, and no recurrence of thoracic hematoma on chest CT.

## Discussion and conclusions

In this report, we present a rare case of RIMA branch perforation during the TRA procedure. An iatrogenic right internal mammary artery perforation occurred during a coronary angiography operation, resulting in a giant right thoracic hematoma. Timely sealing of the perforated vessel helped stabilize the patient. Regarding the hematoma and degenerative heart valve disease, the patient was subjected to valve replacement, ascending aortic replacement and right thoracic hematoma removal in cardiac surgery. Overall, the surgical procedures went smoothly and the patient recovered well.

The TRA is currently the mainstay of percutaneous coronary intervention. A large body of clinical evidence has shown that the complications associated with the TRA are less frequent and less severe than those associated with the femoral artery, and are mostly confined below the elbow [5]. Despite its low incidence, TRA is associated with life-threatening complications. Possible severe complications include subclavian artery entrapment and perforation of the cephalobrachial, thyroid carotid or internal mammary artery [1, 6]. Regarding perforation, Luo et al. found that most of the hematoma in the thorax were related to hydrophilic guidewires during transradial cardiac catheterization [7]. Theoretically, hydrophilic guidewires can possibly cause perforation or hematoma in any branch of the passing artery.

Upon further review of the reported cases, most internal mammary artery perforations occurred during the PCI manipulation of the left internal mammary artery LIMA graft, which is out of the scope of this study. We identified 2 RIMA branch ruptures during transradial access. In the first case, the trajectory was misinterpreted and further catheter manipulation perforated the RIMA during a neuroendovascular procedure [8]. Likewise, the patient developed a large chest wall hematoma and underwent embolization with coils and glue [8]. Ersan Tatli et al. reported a large hematoma in the right breast caused by perforation of the RIMA following transradial coronary angiography, which was managed with implantation of a handmade covered stent [9].

Other related cases of rare deep vascular complications of TRA are summarized in Table 1. Intriguingly, Choi S et al. reported a case of a life-threatening mediastinal hematoma by a hydrophilic wire [10]. During PCI, the patient quickly presented with voice change, dyspnea, and lip cyanosis. The terminal branch of the inferior thyroid artery was ruptured based on brachiocephalic angiography. Interventional occlusion and mediastinoscopic hematoma aspiration were performed, and the patient eventually recovered. Occasionally, the hematoma was visible and protruded the body surface, facilitating appropriate management. As such, Ajay Sharma et al. reported a case of right axillary artery branch perforation via TRA, resulting in a large right chest wall hematoma, and the patient recovered after autologous clot blocking [11]. As mentioned in our case and others, most penetrations did not result in specific symptoms and signs, which delayed the recognition. Prompt bedside imaging and laboratory tests might help decipher possible complications.

**Table 1:** The clinical features of the complications reported during transradial access angiography

Authors,Year	Age	Sex	Arteries Involved	Types of damage	Causation	Hematoma site	Treatment
Shi, 2022 [15]	59	Male	Root of the RSC	Perforation	NA	Right neck and right supraclavicular area	Covered stent
Abecassis, 2021 [8]*	87	Male	Branch of the RIMA	Perforation	Terumo Angled Glidewire	Pectoral hematoma and hemothorax	Coil and glue embolization
Choi, 2020 [10]	57	Male	Branches of the RITA	Perforation	J-type hydrophilic wire	Mediastinal hematoma, and hemothorax	Embolization with coil, gel sponge, and glue
Li, 2019 [16]	54	Male	Proximal RSC	Perforation	Hydrophilic J-wire	Lower right neck and anterior mediastinum	Protamine and conservative treatment
Ghori, 2019 [17]	61	Male	Thyrocervical trunk	Pseudoaneurysm, dissection and perforation	Terumo Runthrough NS Extra Floppy wire	Right neck	Coil and vascular plug embolization
Smilowitz, 2018 [18]	69	Male	Brachiocephalic artery	Pseudoaneurysm	Guide catheter	Anterior mediastinum	Covered stent
Sharma, 2017 [11]	53	Male	Branch of the axillary artery	Perforation	Hydrophilic wires	Right pre-pectoral soft tissue	Conservative treatment
Merkle, 2017 [19]	73	Female	RSC	Perforation	Polymer-jacketed guidewire	Mediastinum	Conservative treatment
	73	Male	Brachiocephalic trunk and AOAR	Dissection	Guide catheter	NA	Conservative treatment
Tatli, 2014 [9]	72	Female	RIMA	Perforation	Terumo Angled Glidewire	Right breast	Handmade covered stent graft
Parikh, 2013 [20]	84	Male	Branch of the SC	Possible perforation	Terumo Angled Glidewire	Left hypopharynx down to upper mediastinum	Conservative treatment
Farooqi, 2013 [21]	81	Female	Right costocervical artery	Pseudoaneurysm and perforation	J-tip guide wire	Upper mediastinum and neck	Coil embolization
Abdool, 2013 [22]	86	Female	RSC	Perforation	NA	Mediastinum	Covered stent graft
Villanueva-Benito, 2012 [23]	43	Male	SC	Pseudoaneurysm	NA	NA	Covered self-expanding stent
Seubert, 2012 [24]	86	Male	RITA	Perforation	Guide wire	Mediastinum	Conservative treatment
Habib, 2012 [25]	79	Female	RSC	Perforation	NA	Mediastinum	Covered stent
Park, 2008 [26]	61	Male	NA	NA	NA	Mediastinum	Conservative treatment
	69	Male	Thymic branch of INA	Perforation	Guide wire	Anterior mediastinum	Histoacylate embolization
Jao, 2003 [27]	57	Male	INA, probably	NA	NA	Superior mediastinum, para-aortic and precarinal area	Conservative treatment

* TRA was used for a neuroendovascular procedure. Abbreviation: AOAR, aortic arch; NA, not available; INA, innominate artery; RITA, right inferior thyroid artery; RIMA, right internal mammary artery; RSC, right subclavian artery; SC, Subclavian artery

Timely detection and management of perforated vessels is very important for patient prognosis. The corresponding measures include covered stent implantation or branch vessel embolization. Covered stents have been commonly used for larger diameter arterial perforations, for example in the management of interventional complications of the internal mammary artery. Eiji Ichimoto and Remo Albiero applied a polytetrafluoroethylene-covered stent to successfully seal the perforation of the LIMA bridge vessel during interventional treatment [12, 13]. Ersan Tatli used a handmade covered stent to seal the RIMA perforation caused by the guidewire [9]. For branch vessel perforation, angioembolization was suggested to be effective. Currently, the embolization materials include spring coils, gelatin sponges, and autologous clotting blocks. A single-center, 12-year study demonstrated that transcatheter coil embolization (TCE) is safe and effective in the treatment of different coronary circulation abnormalities including coronary fistula, LIMA bridge branches, coronary perforation, coronary aneurysm or pseudoaneurysm [14]. Its overall success rate was 87.8%, and no serious TCE-related complications were observed at long-term follow-up. In the two cases of Choi S and Abecassis, spring coils combined with gelatin sponge successfully embolized the perforated arterial branch to prevent the hematoma expansion [8, 10]. In our case, prompt sealing of the arterial breach with a spring coil helped alleviate the bleeding and stabilize the vital signs.

TRA-related thoracic hematoma can cause serious compression to neighbor organs, which, if severe, or not managed appropriately, can require surgical intervention. In Choi S’s case, the patient was subjected to mediastinoscopic hematoma aspiration to eliminate the suffocation [10]. In our case, lung function deteriorated due to compression of the large thoracic hematoma. The drainage partially reduced the encapsulated hematoma volume. The hematoma might not be further excised if not for valve and ascending aorta replacement surgeries.

Although TRA reduced the risk of bleeding and vascular complications compared with transfemoral access, vascular perforation and subsequent hematoma might occur due to guidewire maneuvering. Awareness of prevention, early recognition and management of access complications may help reduce the occurrence and severity of complications related to TRA.

## Data Availability

All data supporting the conclusions are presented in the manuscript.

## List of abbreviations

CAG: coronary angiography
CPB: cardiopulmonary bypass
CT: Computed Tomography
FEV1: Forced expiratory volume in one second
FVC: Forced vital capacity
PCI: percutaneous coronary intervention
RIMA: right internal mammary artery
RSC: right subclavian artery
TRA: transradial approach
TTE: Transthoracic echocardiography
